# Genetic polymorphism of merozoite surface protein-1 and merozoite surface protein-2 in *Plasmodium falciparum* isolates from children in South of Benin

**DOI:** 10.1051/parasite/2013039

**Published:** 2013-10-21

**Authors:** Aurore Ogouyèmi-Hounto, Dorothée Kinde Gazard, Nicaise Ndam, Elsa Topanou, Olivia Garba, Pancras Elegbe, Tatiana Hountohotegbe, Achille Massougbodji

**Affiliations:** 1 Unité d’Enseignement et de Recherche en Parasitologie-Mycologie de la Faculté des Sciences de la Santé 01BP188 Cotonou Bénin; 2 Institut de Recherche pour le Développement 08BP841 Cotonou Bénin; 3 Laboratoire du Centre de Lutte Intégrée contre le Paludisme 01BP188 Cotonou Bénin

**Keywords:** Benin, Genotyping, Merozoite surface protein, *Plasmodium falciparum*

## Abstract

The aim of this study was to determine the genetic diversity of *Plasmodium falciparum* by analyzing the polymorphism of the *msp-1* and *msp-2* genes and the multiplicity of infection in children with uncomplicated malaria in southern Benin. Blood samples of children with fever or history of fever with thick smear positive *P. falciparum* were collected on filter paper. After extraction of DNA by Chelex®, the samples underwent nested PCR. 93 isolates from children were genotyped. For the *msp-1* gene, the K1 and R033 sequences were the most represented in the study population with 85.2% and 83% prevalence, respectively. Regarding the *msp-2* gene, the FC27 family was more highly represented with 99% prevalence against 81.5% for 3D7. Mixed infections accounted for 80.4% of the samples. Twenty-five alleles were identified for *msp-1* and 28 for *msp-2*. Fourteen and ten alleles belonged to the K1 (100–500 bp) and MAD20 (100–500 bp) families, respectively. The RO33 sequence did not show any polymorphism, with only one variant (160 bp) detected. The *msp-2* gene was present as 16 FC27 family fragments (250–800 bp) and 12 of the 3D7 family (350–700 bp). The multiplicity of infection was estimated at 3.8 for *msp-1* and 3.9 for *msp-2* with 77 (87.5%) and 84 (91.3%) samples harboring more than one parasite genotype for *msp-1* and *msp-2*, respectively. The multiplicity of infection (MOI) was influenced neither by age nor by parasite density. This study shows a significant diversity of *P. falciparum* in southern Benin with an MOI unaffected by age or by parasite density.

## Abstract (fr)

Le but de cette étude était de déterminer la diversité génétique de *Plasmodium falciparum* en analysant le polymorphisme des gènes *msp-1* et *msp-2* et la multiplicité de l’infection chez les enfants souffrant de paludisme simple au sud du Bénin. Les enfants présentant une fièvre ou antécédents de fièvre avec une goutte épaisse positive à *P. falciparum* ont subi un prélèvement de sang sur papier buvard. Après extraction de l’ADN au Chelex®, les échantillons ont fait l’objet d’une PCR nichée. Les isolats de 93 enfants ont été génotypés. Pour le gène *msp-1*, les familles K1 et R033 étaient les plus représentées dans la population d’étude avec respectivement 85,2 % et 82,9 %. Pour *msp-2*, la famille FC27 était la plus représentée avec 98,9 % contre 81,6 % pour 3D7. Les infections mixtes représentaient 80,4 % des échantillons. Vingt-cinq allèles ont été identifiés pour *msp-1* et 28 pour *msp-2.* Quatorze et dix allèles appartenaient respectivement à la famille K1 (100–500 bp) et MAD20 (100–500 bp). Aucun polymorphisme n’a été noté avec la famille RO33, avec un seul fragment (160 bp) détecté. Le gène *msp-2* présentait 16 fragments pour FC27 (250–800 bp) et 12 pour 3D7 (350–700 bp). La multiplicité de l’infection était de 3,8 pour *msp-1* et 3,9 pour *msp-2* avec 77 (87,5 %) et 84 (91,3 %) échantillons qui avaient plus d’un génotype parasitaire respectivement pour *msp-1* et *msp-2*. La multiplicité de l’infection (MOI) ne variait pas significativement avec l’âge ni avec la densité parasitaire. Cette étude montre une importante diversité de *P. falciparum* au sud du Bénin avec une MOI qui n’est influencée ni par l’âge ni par la densité parasitaire.

## Introduction

In Benin, *P falciparum* infection is among the first diseases and is also responsible for 36% of deaths among children under 5 years (unpublished data from Ministry of Health). Despite the enormous efforts that have been directed toward malaria control and prevention, multiple factors including insecticide resistance in the anopheline vectors, and the emergence and rapid spread of drug-resistant parasite strains are major problems for the control and prevention of malaria. Therefore, the development of an effective malaria vaccine is urgently needed. One of the limitations to the development of this vaccine against *P. falciparum* is the extensive genetic diversity in parasite populations limiting the efficacy of acquired protective immunity to malaria [6]. Indeed, antigenic diversity is one of the hypotheses advanced to explain the slow acquisition of immunity against malaria in individuals living in malaria-endemic areas [15]. Asexual blood stage antigens, such as merozoite surface protein-1 (*msp-1*) and merozoite surface protein-2 (*msp-2*), are considered prime candidates for the development of a malaria vaccine and are also suitable markers for the identification of genetically distinct *P. falciparum* parasite sub-populations [5]. In Benin a study performed by Issifou *et al*. [9] noted a high prevalence of multiple infections influenced neither by age nor by season in a group of children and adults with uncomplicated malaria. Since then, no other study has explored the genetic diversity of *P. falciparum* malaria in Benin. Following scale up of malaria control interventions (massive deployment of insecticide-treated nets and free treatment with ACT) in the country, it is important to reassess genetic diversity of *P. falciparum* in Benin to better direct control actions. This study aimed to characterize the allelic polymorphism of *msp-1* and *msp-2* and determined the multiplicity of infection in *P. falciparum* isolates collected from children with uncomplicated malaria living in the southern Benin.

## Patients and methods

### Study sites and population

The study was conducted in two highly endemic regions of southern Benin, including the departments of Littoral and Ouémé. Southern Benin is characterized by a sub-equatorial climate and a perennial malaria transmission with two peaks corresponding to the rainy seasons (April–July and mid-September–November) [20]. Children aged 6 months to 15 years who resided in the study sites for more than a period of 6 months were enrolled from May through August 2011. Children visiting the health facilities in the study area and who met the criteria below were enrolled in the study: (i) fever (axillary temperature ≥37.5 °C) or a history of fever within the past 48 h; (ii) *P. falciparum* mono-infection with parasite density ≥1000 asexual forms per microliter, identified by microscopy on blood smears; (iii) no evidence of a concomitant febrile illness; (iv) no sign/symptoms of severe malaria as defined by WHO [28]; and (v) written informed consent from parents.

### Sample collection and laboratory procedures

Venous blood from symptomatic children fulfilling the above criteria was collected systematically on the filter paper. Thick and thin blood smears were prepared and were stained with 10% Giemsa for rapid diagnosis. All thick blood smears were examined against 500 leukocytes. Parasite densities were recorded as the number of parasites/μL of blood, assuming an average leukocyte count of 8000/μL of blood. All slides were read in the health center’s laboratory with external quality control performed on 10% of the negative slides and all positives in the Reference Laboratory of Parasitology of the Centre National Hospitalo Universitaire of Cotonou. Parasite DNA was extracted from filter papers using the Chelex 100 resin methods [19] and stored at −20 °C until use.

### Molecular genotyping of the polymorphic genes *msp-1* and *msp-2*

Specific primer pairs were used to amplify block 2 of *msp-1* and block 3 of *msp-2* [16, 23]. The two genes were amplified by nested PCR, each amplification with conserved or family specific primer pairs, being done separately, as described previously [25]. Analyses of the K1, MAD20, and RO33 allelic families of *msp-1* and the *3D7* and FC27 allelic families of *msp-2* were performed sequentially in accordance with the genotyping protocol of Snounou *et al*. [25]. Allele-specific positive controls and DNA free negative controls were included in each set of reactions. Gel photographs were re-scored by visual comparison of DNA fragments and for individual samples, alleles were identified according to band size and the corresponding allele-specific primers used. The size of the PCR products was estimated using a 100 bp DNA ladder marker (Boehringer Mannheim, Marker VI).

### Data analysis

The data were entered in the software R version 2.12.0 (R Foundation for Statistical Computing, Vienna, Austria). The distribution of allelic families of *msp-1 and msp-2* genes was determined by the number of PCR products corresponding to each family within the total number of samples. The number of patients with more than one amplified PCR fragment within the total population is defined as the frequency of polyclonal infections. The multiplicity of infection (MOI) was calculated as the total number of detected *Plasmodium falciparum msp-1, msp-2* genotypes/total number of infected children [12]. Student’s test was used to compare MOI. The chi-square test or Fisher’s exact test was used for proportion comparisons. The *p* value < 0.05 was chosen as threshold significance for the various statistical tests.

### Ethics statement

The Ethical Committee of the School of Medicine and Health Sciences, University of Benin, gave the ethical approval for the study. Written informed consent from head of school and parents was received.

## Results

### Demographic and parasitological data of the study population

Over a period of 4 months, 93 children meeting the inclusion criteria were recruited to the study. The characteristics of the study population are detailed in Table 1. The children’s ages ranged from 6 months to 15 years (mean age: 7.9 ± 0.4 years). The parasite density ranged from 1,000 to 160,000 parasites/μL with a mean density of 19,093.

**Table 1.** Demographic and parasitological data of the study population.

Characteristics of patients	Values
Mean age (year)	7.9 ± 0.4
Age range	6 months to 15 years
Sex ratio (M/F)	1.3 (53/40)
Geometric mean of parasitemia	19,093 [12,698; 28,707]
Parasite density range (p/μL)	1,000–160,000

The parasite DNA from the 93 *P. falciparum* isolates was analyzed for *msp-1* and *msp-2* genes. The efficiency of *msp-1* and *msp-2* gene amplification reactions with family-specific primers was 94.6% (88/93) and 99% (92/93), respectively.

### Genetic diversity of *P. falciparum msp-1* and *msp-*2 gene

The distribution of the different allelic families of *msp-1* and *msp-2* genes is shown in Table 2. The K1 family was the predominant allelic type among mixed infections with RO33 (20.5%) and most represented in the overall population (85.2% prevalence) following by the RO33 sequence (83%) without significant difference *p* > 0.05. For the *msp-2* gene, the FC27 family was more represented but the difference was not statistically significant (*p* = 0.57).

**Table 2.** Genetic diversity of *P. falciparum.*

Family	*N* (%)	Family	*N* (%)
*MSP1*	*n* = 88	MSP2	*N* = 92
K1	5 (5.7)	FC27	17 (18.5)
MAD20	3 (3.4)	3D7	1 (1.1)
RO33	6 (6.8)	3D7 + FC27	74 (80.4)
K1 + MAD20	7 (8)	Total FC27	91 (98.9)
K1 + RO33	18 (20.5)	Total 3D7	75 (81.5)
MAD20 + RO33	4 (4.5)	Multiples infection	84 (91.3%)
K1 + MAD20 +RO33	45 (51.1)	MOI	3.9
Total K1	75(85.2%)		
Total RO33	73 (82.9%)		
Total MAD20	59 (67%)		
Multiples infection	77 (87.5%)		
MOI	3.8		

The number of *msp-1* and *msp-2* genotypes per isolate ranged from 1 to 9. The MOI was 3.8 ± 0.35 for *msp-1* and 3.9 ± 0.30 for *msp-2.* Multiple infections were found in 87.5% (77/88) of samples for *msp-1* and 91.3% (84/93) of samples for *msp-2*. Overall (*msp-1 + msp-2*), the multiplicity of infection was 4 PCR fragments per individual infected, with 72 (81.8%) of the samples harboring more than one parasite genotype.

According to age, and parasite density the MOI was similar between individuals of different age and parasite density without significant difference (Table 3).

**Table 3.** MOI according age and parasite density.

	MOI		
Age	*msp-1*	*msp-2*	*msp-1 + msp-2*
≤5 years	3.9	3.8	4.0
5–10 years	3.7	4.0	3.9
≥10 years	3.8	3.9	4.2
*p* Value	*p* > 0.05	*p* > 0.05	*p* > 0.05
Parasite density			
≤5000	3.6	3.4	4.2
5001–10 000	3.5	3.9	3.8
≥10 000	3.8	3.7	4.0
*p* Value	*p* > 0.05	*p* > 0.05	*p* > 0.05

A total of 53 individual *msp* alleles were identified (25 for *msp-1* and 28 for *msp-2*).

**Figure 1.**
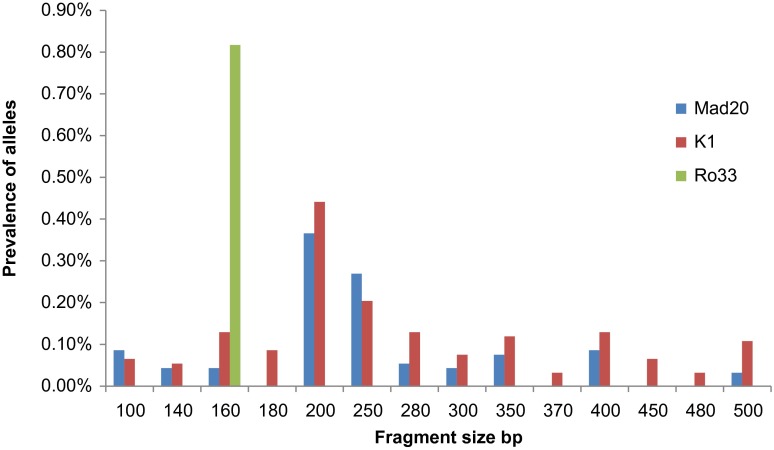
Prevalence of *P. falciparum* K1, RO33 and MAD20 *msp-1* alleles classified by length (in base pairs).

**Figure 2.**
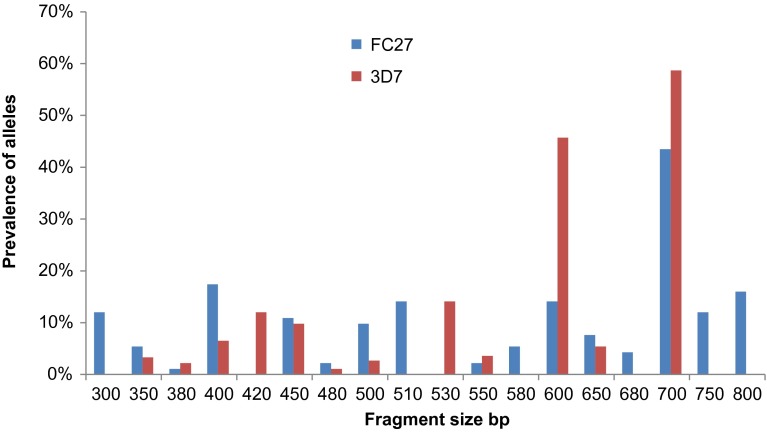
Prevalence of *P. falciparum* FC27 and 3D7 *msp-2* alleles classified by length (in base pairs).

For the *msp-1* gene, 14 K1 type alleles (100–500 bp); 1 RO33-type allele (160 bp) and 10 MAD20 type alleles (100–500 bp) were identified; for the *msp-2* gene 16 different FC27 type alleles (250–800 bp) and 12 3D7 type alleles (350–700 bp) were detected. The analysis of K1 alleles revealed three prevalent alleles (200 bp, 250 bp, and 400 bp PCR fragments). The RO33 allele did not show any polymorphism, with only one variant (160 bp). The most prevalent Mad20 type alleles were represented by the Mad20_200_ and Mad20_250_ PCR fragments (Figure 1). For the *msp-2* gene, the most prevalent alleles were detected by the FC27_700_, 3D7_600_, and 3D7_700_ PCR fragments (Figure 2).

## Discussion

In the Republic of Benin, less attention has been put on the investigation of the genetic diversity of *P. falciparum* than elsewhere. This study is the second after that conducted by Issifou in 2001 [9]. with intensification of malaria control (scale up of use of insecticide-treated bed nets (ITNs), intermittent preventive treatment to pregnant women, early and obligatory parasitological diagnosis before treatment of clinical cases using artemisinin-based combination therapy (ACT)), it was important to characterize new strains of *Plasmodium falciparum* found in Benin through the importance of mixed infections and the multiplicity of infections. This study was also important for the country because diversity may influence the degree and the expansion of parasite resistance to antimalarial drugs. A high degree of polymorphism is the basis of a large chromosomal recombination thereby increasing the prevalence of the resistance gene. The purpose of this study was to determine the genetic diversity of *P. falciparum* using the two most polymorphic regions of *msp-1* and *msp-2*, in malaria symptomatic subjects in southern Benin.

Allele-specific genotyping of *msp-1* and *msp-2* showed a high genetic diversity in the *P. falciparum* population studied in southern Benin. The degree of polymorphism found in the present study is also consistent with the high level of malaria transmission in the study area as reported previously [22] with a higher diversity of *msp-2* in agreement with other studies [1, 21, 24]. Twenty-five alleles of *msp-1* were observed, in which, the K1 allelic family was predominant, consistent with most previous studies [1, 9, 26] but in contrast with one in India [2] showing that the RO33 allele was predominant. The limited polymorphism in the RO33 alleles is similar to the findings of others in African countries [14, 17]. For the *msp-2* locus, 28 alleles were found and alleles belonging to FC27 family were most frequently detected. Although this is similar to data reported for a study in Congo [12], it differs from the previous results of Issifou in Benin [9], and Mayengue in Congo Brazzaville [14]. The difference with the results of Issifou may be due to the fact that the study goes far back in the past. Indeed, according to Yuan in Myanmar [29], the majority of alleles showed significant temporal fluctuations through the years. Some genotypes underwent major fluctuations in density, while others were highly stable. It has been shown that parasite polymorphism is poorly evaluated by examination of a single blood sample because genotypes can appear and disappear in a very short time [4, 7]. This occurred faster in the higher transmission areas when compared to lower transmission areas. In the same context, Mayengue *et al*. [13] had found evidence of intra and inter-individual variation in the number of parasite genotypes present in the different episodes of malaria. These results suggest the need for several studies on several samples in the same region to assess the genetic profile of parasites. In our study, a limited clonal fluctuation to a maximum of three predominant alleles was found, similar to the results observed in Burkina Faso [26] as well as in other countries of low endemicity, and with low polymorphism [3, 8]. The high rate of multiple or mixed *P. falciparum* infections with *msp-1* and *msp-2* also found elsewhere [12, 26] would probably be a consequence of high malaria transmission in the study areas where individuals are exposed to frequent mosquito bites and therefore a significant inoculation of parasite populations that are genetically different. Therefore, these results suggest that the degree of transmission affects the occurrence of mixed infections as initially demonstrated in the Snewin study [24]. Actually, the mean MOI of 3.8 (*msp-1*) and 3.9 (*msp-2*) was high compared to those reported in Benin and Congo Brazzaville [9, 14] but consistent with those reported from Gabon, Burkina Faso, and Senegal [1, 26, 27]. This high rate of MOI in the present study suggests that despite the intensification of malaria control interventions involving reduction of malaria infection, the parasite population size and transmission intensity has remained high enough to allow effective genetic recombination of the parasites and continued maintenance of genetic diversity. This echoes the Bogreau study which showed that there is no variation of genetic diversity in a population before and after the use of ITNs.

The fact that the MOI was not influenced by age as shown in other countries [11, 18] suggests that the MOI is not directly related to the period of acquisition of immunity in asymptomatic children, but reflects the exposure of subjects to malaria in the endemic area. Thus, a study of the multiplicity of infection and immunity against asexual stages of Plasmodium [12] showed that the samples with the highest multiplicity came from children with significantly lower antibody responses to specific antigens of the asexual parasite. Malaria episodes with many clones would reflect a low level of acquired immunity with consequent limited ability to control the infection. For these authors, the multiplicity of infection with *P. falciparum* could be a potentially useful parameter in the evaluation of interventions against malaria. Several studies have shown a correlation between the MOI and parasite density, but in our study, the MOI did not increase with higher densities similar to results of other studies conducted elsewhere [10, 18].

## Conclusion

These results show a high genetic diversity of populations of *P. falciparum* isolated in southern Benin. This study has demonstrated once again the link between the high level of transmission, mixed infections and multiplicity of infection already found in other countries in the subregion and elsewhere. The MOI is not influenced either by patient age or by parasite density. However, other longitudinal studies to examine the dynamics of the genetic diversity of *P. falciparum,* taking into account other factors such as the degree of transmission, the study of the immune response, and studies of genes conferring resistance to antimalarial drugs, are needed to better target malaria control.
